# Cerebral Cell Renewal in Adult Mice Controls the Onset of Obesity

**DOI:** 10.1371/journal.pone.0072029

**Published:** 2013-08-13

**Authors:** Alexandra Gouazé, Xavier Brenachot, Caroline Rigault, Alice Krezymon, Camille Rauch, Emmanuelle Nédélec, Aleth Lemoine, Jean Gascuel, Sylvian Bauer, Luc Pénicaud, Alexandre Benani

**Affiliations:** 1 Centre des Sciences du Goût et de l’Alimentation, Centre National de la Recherche Scientifique - Institut National de la Recherche Agronomique - Université de Bourgogne, Dijon, France; 2 Institut de Neurobiologie de la Méditerranée, Institut National de la Santé et de la Recherche Médicale, Marseille, France; Institut National de la Recherche Agronomique-CNRS UMR6175, France

## Abstract

The hypothalamus plays a crucial role in the control of the energy balance and also retains neurogenic potential into adulthood. Recent studies have reported the severe alteration of the cell turn-over in the hypothalamus of obese animals and it has been proposed that a neurogenic deficiency in the hypothalamus could be involved in the development of obesity. To explore this possibility, we examined hypothalamic cell renewal during the homeostatic response to dietary fat in mice, i.e., at the onset of diet-induced obesity. We found that switching to high-fat diet (HFD) accelerated cell renewal in the hypothalamus through a local, rapid and transient increase in cell proliferation, peaking three days after introducing the HFD. Blocking HFD-induced cell proliferation by central delivery of an antimitotic drug prevented the food intake normalization observed after HFD introduction and accelerated the onset of obesity. This result showed that HFD-induced dividing brain cells supported an adaptive anorectic function. In addition, we found that the percentage of newly generated neurons adopting a POMC-phenotype in the arcuate nucleus was increased by HFD. This observation suggested that the maturation of neurons in feeding circuits was nutritionally regulated to adjust future energy intake. Taken together, these results showed that adult cerebral cell renewal was remarkably responsive to nutritional conditions. This constituted a physiological trait required to prevent severe weight gain under HFD. Hence this report highlighted the amazing plasticity of feeding circuits and brought new insights into our understanding of the nutritional regulation of the energy balance.

## Introduction

New neurons are continuously produced in the adult mammalian hypothalamus [1]. The ependymal layer surrounding the third ventricle and the median eminence form delimited neurogenic niches [2], [3], [4], previously reported to occur in the ventricular zone at all points along the neuraxis [5]. The hypothalamic parenchyma also contains scatered mitogenic activity supporting the generation of new functional neurons [6], [7]. However, the physiological functions of these newly generated neurons or of neuronal progenitors in the adult hypothalamus remain uncertain. Nonetheless, the mitotic activity of hypothalamic progenitors is modulated by changes in environmental and physiological conditions [3], [8], [9], [10], [11]. It could also be artificially stimulated by the administration of growth factors or hormones, thus being candidates as endogenous mediators of such changes [2], [4], [12], [13]. Hence, the constitutive neurogenesis in the adult hypothalamus could play a role in the maintenance of the long-term stability of the inner milieu and could serve as an adaptive reservoir that finely adjusts physiological processes to long-lasting environmental changes.

Centrally administered ciliary neurotrophic factor (CNTF) induces cell proliferation in several parts of the mouse brain including the hypothalamus. This proliferative effect is required for the long-term anti-obesity effect of CNTF suggesting that the hypothalamic neurogenesis in adult mice could play a role in energy balance [12]. Indeed, under progressive neurodegeneration, orexigenic neurons of the hypothalamus spontaneously regenerate to preserve the integrity of feeding circuits and to maintain normal eating behavior [7]. The continuous turn-over of neurons located in the arcuate nucleus of the hypothalamus is suppressed in obese mice [11] suggesting that the lack of remodeling in feeding circuits could contribute to the development of obesity. On the other hand, cell proliferation in the median eminence of the hypothalamus supports energy storage during high-fat diet (HFD) [3]. This suggests that the hypothalamic neurogenesis exerts different functions depending on its location.

A better understanding of the nutritionally-regulated turn-over and maturation of newborn neurons in the adult hypothalamus could help in finding novel therapeutic strategies to fight against overweight, dramatically rising in the world and constituting a major risk factor for a number of chronic diseases, such as diabetes and cardiovascular diseases. Although the severe alteration of cell turn-over in the hypothalamus of obese animals is now well documented [11], [14], the changes that occurs early upon HFD administration, i.e. before the apparition of obesity, are not known. Moreover, the degree to which hypothalamic cell renewal participates in the regulation of energy homeostasis in non-obese conditions remains unclear. Thus, we performed a quantitative mapping of hypothalamic cell renewal in mice, at the onset of diet-induced obesity. We observed that HFD rapidly stimulated cell proliferation in the hypothalamus. We also assessed the functional role of cerebral cell renewal in energy balance after a pharmacological block of cell proliferation in the whole brain. We observed that proliferative cerebral cells were crucial for the maintenance of body weight. Moreover, analysis of neuronal subtype markers of newly generated cells revealed that HFD altered the fate of newborn neurons in the hypothalamus, probably as an homeostatic mechanism for long-term regulation of food intake.

## Methods

### Animals

All protocols involving animals were reviewed and approved by our local Institutional Animal Care and Use Committee (“Comité d’éthique de l’expérimentation animale de l’Université de Bourgogne”; agrement number B1310), and were in strict accordance with European Community guidelines (directive 86/906). Seven week-old male C57BL/6 mice were obtained from Harlan Laboratories and housed one per cage at 21–22°C on a 12/12-hour light/dark cycle (light on at 08h00). Mice were acclimated for one week before the initiation of experiments. They had free access to food and water. Food was a standard pelletized rodent diet (#A04; Safe) or high-fat diet (HFD) (customized semi-synthetic food; Safe; see [15]). Food was daily renewed at 09h00.

### Intracerebroventricular Drugs Administration

Intracerebroventricular (icv) cannulation and osmotic minipump implantation were performed at the same time. Under isoflurane anesthesia, mice were stereotactically implanted with a cannula (Brain infusion kit III; Charles River) into the right lateral ventricle (anteroposterior −0.2 mm and lateral +1.0 mm to Bregma; dorsoventral −1.9 below skull). The cannula was connected to an osmotic minipump (model 1007D for 1 week infusion or less, model 2004 for 1 month infusion or less; Charles River) via vinyl tubing (inner diameter 1.22 mm; Alzet) filled with vehicle (NaCl 9‰; Sigma) as fully previously described by Kokoeva et al. [12]. The 65 mm length of tubing allowed 2 days of vehicle infusion after the surgery. This time was sufficient for the minipump content to reach the ventricular system. Minipumps were filled either with vehicle, bromodeoxyuridine (BrdU, 1 µg/µl in vehicle; Sigma), arabinofuranosyl cytosine (AraC, 6.66 µg/µl in vehicle; Sigma), or a mix of BrdU and AraC. Before implantation, minipumps were primed overnight at 37°C in NaCl 9‰. After surgery, mice recovered normal food intake within 48 hours (≈ 0.5 kcal/d/g of body weight). Starting time of experiments (T0, e.g. HFD introduction) was set at 4 days post-surgery.

### Tissue Processing and Reagents for Immunohistochemistry

For histological analysis, mice were anesthetized by intraperitoneal injection of a ketamine/xylazine mix (10 µl/g of mouse; 100/10 mg/kg), and perfused transcardially with 9‰ NaCl containing 250 U Heparin (Sanofi), followed by a fixative solution (4% formaldehyde solution; Sigma). Brains were dissected, immersed overnight in fixative solution, transferred to 30% sucrose (Sigma) for 24 or 48 hours and sectioned with a cryostat (Leica) in the coronal plane (section thickness = 25 µm). Sections were collected in four series (every fourth in one aliquote) in cryoprotectant (30% v/v glycerol, 30% v/v ethylene glycol, 154 mM NaCl, 100 mM Tris-HCl, pH 7.5) and stored at −20°C until use. Free-floating sections were probed using the following primary antibodies and working concentrations: rat anti-BrdU (1∶100; #OBT0030; AbD Serotec), rabbit anti-GFAP (1∶800; #18-0063; Molecular Probe), rabbit anti-Iba1 (1∶4000; CP 290A; Biocare Medical), mouse anti-Ki67 (1∶400; 550609; BD Biosciences), mouse anti-NeuN (1∶100; MAB377; Chemicon), rabbit anti-POMC precursor (1∶4000; H-029-30; Phoenix Pharmaceuticals). Fluorescent stainings were performed using Alexafluor 488 goat anti-mouse (1∶500; #A-11029; Molecular Probe), Alexafluor 488 goat anti-rabbit (1∶500; A-11034; Molecular Probe), Alexafluor 555 goat anti-mouse (1∶250; #A-21424; Molecular Probe), or Dylight649 donkey anti-rat antibody (1∶250; #712-496-153; Jackson Immunoresearch). Ki67 detection was made using biotinylated goat anti-mouse antibody (1∶200), peroxidase-conjugated avidin and diaminobenzidine using the Vectastain® ABC kit (Vector Laboratories).

### Antigen Unmasking Procedure and Immunostaining

For BrdU detection, sections were first incubated in 50% formamide/2×SSC at 65°C for 2 hours, rinsed in 2×SSC at room temperature (RT) for 5 minutes, incubated in 2 N HCl at 37°C for 30 minutes, and rinsed in 0.1 M sodium borate (pH 8.5) at RT for 10 minutes. For Ki67 detection, sections were first incubated in citrate solution at 95°C for 10 minutes. For other antigen detection, no specific unmasking procedure was required. All sections were rinsed in PBS (pH 7.4), and blocked with 3% normal goat serum in PBS containing 0.5% Triton X-100 for 1 hour. For POMC neurons labeling, the blocking solution was 3% normal donkey serum in PBS containing 0.5% Triton X-100. Sections were then incubated overnight with primary antibody diluted in the blocking solution at 4°C or at RT for Ki67 labeling. Sections were washed in PBS and then incubated with secondary antibody in the blocking solution for 2 hours at RT. For double-labeling, a two-step procedure was performed. Sections were finally washed in PBS, mounted on SuperFrost® Ultra Plus® slides (Thermo-Fisher Scientific) and coverslipped over Fluorescence Mounting Medium (Dako).

### Imaging and Cell Counting

Stained cells were counted in the whole hypothalamus, from −2.70 mm to +0.58 mm from Bregma, according to a mouse brain atlas [16]. For Ki67 labeling, cells were also counted in three hypothalamic nuclei: the arcuate nucleus (Arc), the ventromedial nucleus (VMN), and the paraventricular nucleus (PVN). To avoid oversampling, every fourth coronal section (25 µm thickness) throughout the hypothalamus was stained and analyzed under a microscope (Axio Imager 2, Zeiss). The entire hypothalamic area was scanned automatically using a motorized stage and acquired at a magnification of ×40 to generate the mosaic image from individual tiles with the Axiovision software and the MosaiX module (Zeiss). Cell counting was performed manually on mosaic images with the assistance of ImageJ software (version 1.46; http://imagej.nih.gov/ij/). To obtain the total cell number of one animal, countings of all affiliated sections were tallied and multiplied by 4 according to the section sampling interval. Double fluorescence-labeled sections were also examined under structured illumination with the Apotome to generate optical sections. To check cases of mere signal superposition, we used the structured illumination microscopy found with the ApoTome microscop setup (Zeiss). ApoTome setup provides an axial resolution in the z-axis that is comparable to that achieved by confocal microscopy [17]. We also examined some samples using laser-scanning confocal microscope (Leica TCS SP2). No noticeable difference in the images generated by either the ApoTome setup or the confocal microscope was found.

### Body Composition Analysis

For the assessment of adiposity, animals were placed in the scanner (EchoMRI-700) after anesthesia by intraperitoneal injection of a ketamine/xylazine mix (10 µl/g of mouse; 100/10 mg/kg) in order to remove stainless steel cannula.

### Tissue Collection

After body composition analysis, animals were sacrificed and dissected to collect brown adipose tissue (BAT), inguinal and retroperitoneal white adipose tissues (subcutaneous and visceral WAT, respectively). BAT was snapfrozen in liquid nitrogen and stored at −80°C until analysis. WAT depots were weighed before further analysis.

### RNA Extraction and Processing

BAT were lysed and homogenized in 1 mL lysis buffer (QIAzol, Qiagen) using the TissueLyser system (Qiagen) and 5 mm stainless steel beads (Qiagen). Total RNAs were extracted using a phenol/chloroform isolation procedure, precipitated with isopropanol washed 3 times with 70% ethanol and resuspended with 50 µl water. Aliquots of each extract were checked for RNA purity and integrity with the Experion electrophoresis system (Bio-Rad Laboratories) and the Experion RNA StdSens Analysis Kit (Bio-Rad Laboratories). RNA concentrations were determined using the Nanodrop spectrophotometer (Thermo Scientific).

### Real-time PCR Analysis

Reverse transcription was performed with 500 ng of total RNA, using the High-Capacity RNA-to-cDNA Master Mix (Applied Biosystem), according to the manufacturer. For qPCR, the Fast SYBR Green Master Mix (Applied Biosystems) was used. Each reaction contained 1 µl of cDNA (diluted 1∶10) and 200 nM of gene specific intron-spanning primers. The sequences of the primers used for ucp1 amplification were 5′-CAGTGTCCAGCGGGAAGGT-3′ and 5′-TCCAAGCCAGGATGGTGAAC-3′. Polr2a was used as an endogenous control. The sequences of the primers were 5′-GCCAAAGACTCCTTCACTCACTGT-3′ and 5′-TGTATGTTCCAAGCGGCAAA-3′. Reaction mixtures were loaded into a 96-well plate, which was placed in a thermal cycler (model Step-One Plus, Applied Biosystems). PCR conditions were 20 s at 95°C, followed by 40 cycles of 3 s at 95°C and 30 s at 60°C. Raw fluorescence data were collected through the PCR using the SDS 2.3 software (Applied Biosystems), which further generated threshold cycles “Ct” with automatic determination of both baseline and threshold. Data were therefore analyzed with RQ Manager 1.2 software (Applied Biosystems) for relative quantitation. Relative quantitation of gene expression (RQ) was based on the comparative Ct method using the equation RQ = 2^−ΔΔCt^, where ΔΔCt for one gene target was its own Ct variation subtracted from a calibrator sample and normalized with an endogenous control. Precisely, polr2a was defined as the endogenous control, and one control sample was arbitrary chosen as a calibrator. After amplification, a melting curve was plotted to check the specific Tm of each PCR product.

### Statistical Analysis

All data are expressed as mean. Error bars indicate standard errors of the mean (SEM). Multiple comparisons of groups were carried out by a one-way analysis of variance (ANOVA) using Prism 4.0 software (GraphPad Software). Post hoc Newman-Keuls test was used to compare groups when main effects reached significance. Unpaired t test was used when two groups were compared. Significant difference was noted *, **, or *** on the graphic representation when p value was <0.05, <0.01, and <0.001, respectively.

## Results

### Rapid Modification of the Constitutive Cell Renewal Rate in the Adult Hypothalamus by HFD

**Figure 1:**
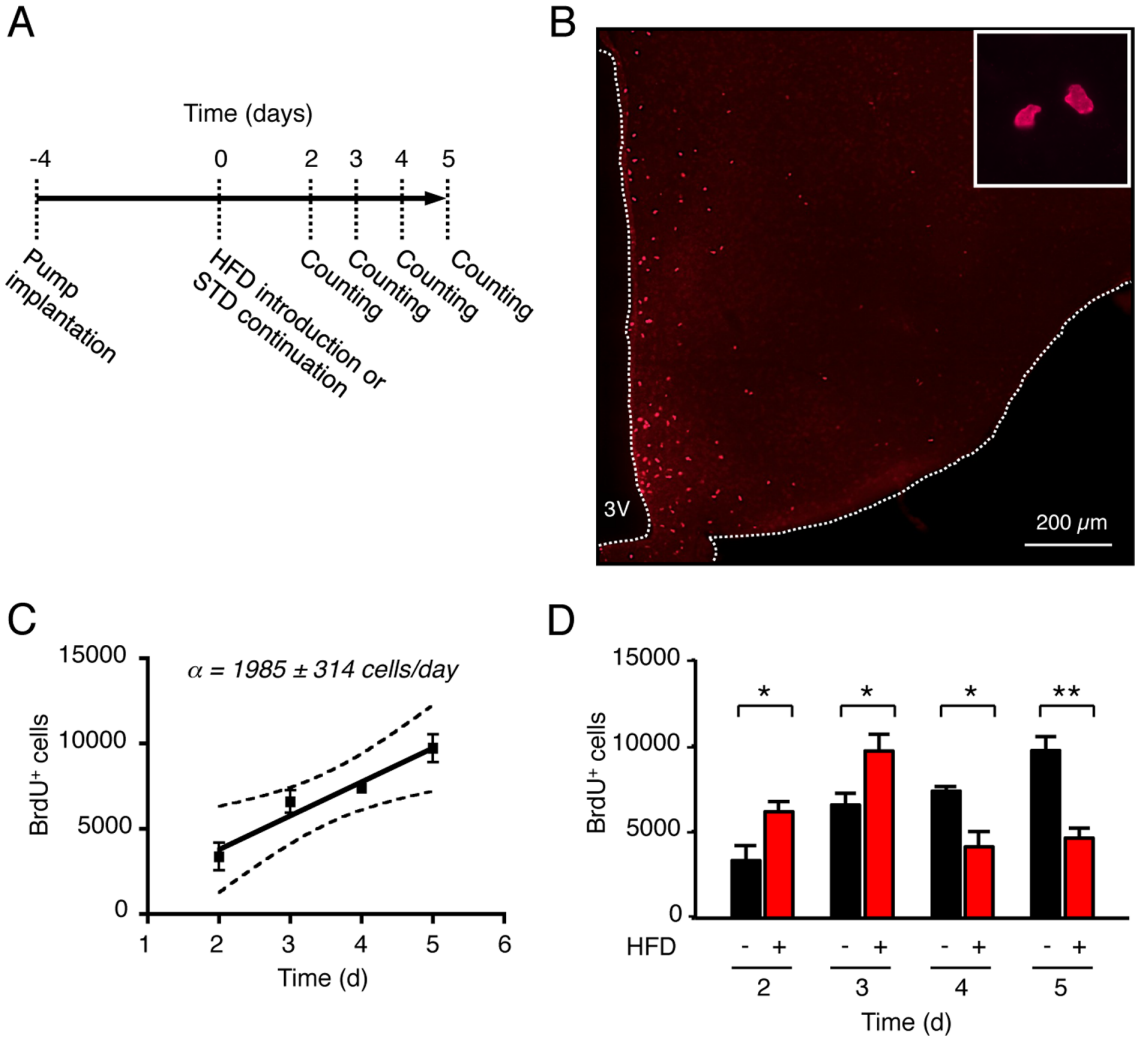
High-fat diet (HFD) caused fast and biphasic variation of cell renewal in the hypothalamus. (A) Schematic representation of the experimental design. Four days before HFD introduction, BrdU-filled osmotic minipumps were implanted subcutaneously and connected to ventricular system to centrally deliver 12 µg/day BrdU (at 0.5 µl/hr). Mice were sacrificed 2, 3, 4 and 5 days after HFD introduction. (B) Representative image of BrdU-labeled newborn cells immunodetected in the hypothalamus of control mice after 5 days. (C) Linear regression modeling the daily rate of BrdU-positive cell accumulation in the whole hypothalamus of control mice (n = 3–5 mice per time-point; r^2^ = 0.952). (D) Quantification of BrdU-positive cells detected in hypothalamus of mice fed either a standard diet or HFD (n = 3–5 mice per group). Data are means ± SEM. Groups were compared using unpaired t test. * and ** denotes p≤0.05 and p≤0.01, respectively. 3V: third ventricle; HFD: high-fat diet.

To detect constitutive cell renewal in the hypothalamus and to explore the effects of HFD on it, we used a very sensitive *in situ* assay of cell proliferation based on the central and continuous delivery of the proliferative marker 5′-bromo-2-deoxyuridine (BrdU), as previously described [6], [12], [13]. Animals were implanted with 1 µg/µl BrdU-filled osmotic minipump connected to a cannula targeting the brain ventricular system. BrdU was infused at a rate of 0.5 µl/h, diffusing through the entire hypothalamic parenchyma after 3 to 4 days [6], [12]. Mice were sacrificed 2, 3, 4 or 5 days after the diffusion period (Fig. 1A) and their brains were collected and processed for BrdU immunohistochemistry (Fig. 1B). In control mice, the number of BrdU-positive cells in the hypothalamus regularly increased over time, from 3374±822 at day 2 to 9726±803 at day 5 (Fig. 1C). Linear regression analysis indicated an increment of 1985±314 cells per day over the analyzed period, confirming the existence of a constitutive cell proliferation in the adult hypothalamus. In HFD mice, the number of BrdU-positive cells in the hypothalamus showed dual variation. Indeed, it was first increased over controls at day 2 (STD: 3374±822 vs HFD: 6178±602; p<0.05) and day 3 (STD: 6594±662 vs HFD: 9706±934; p<0.05) (Fig. 1D), then reduced at day 4 (STD: 7383±249 vs HFD: 4137±908; p<0.05) and day 5 (STD: 9726±803 vs HFD: 4649±559; p<0.01). Thus, HFD caused a rapid and biphasic modification of cell renewal in the murine hypothalamus, with an initial over-production of BrdU-positive cells up to 3 days after HFD introduction, followed by a dramatic 50% reduction.

### Transient Stimulation of Cell Proliferation in the Adult Hypothalamus by HFD

**Figure 2:**
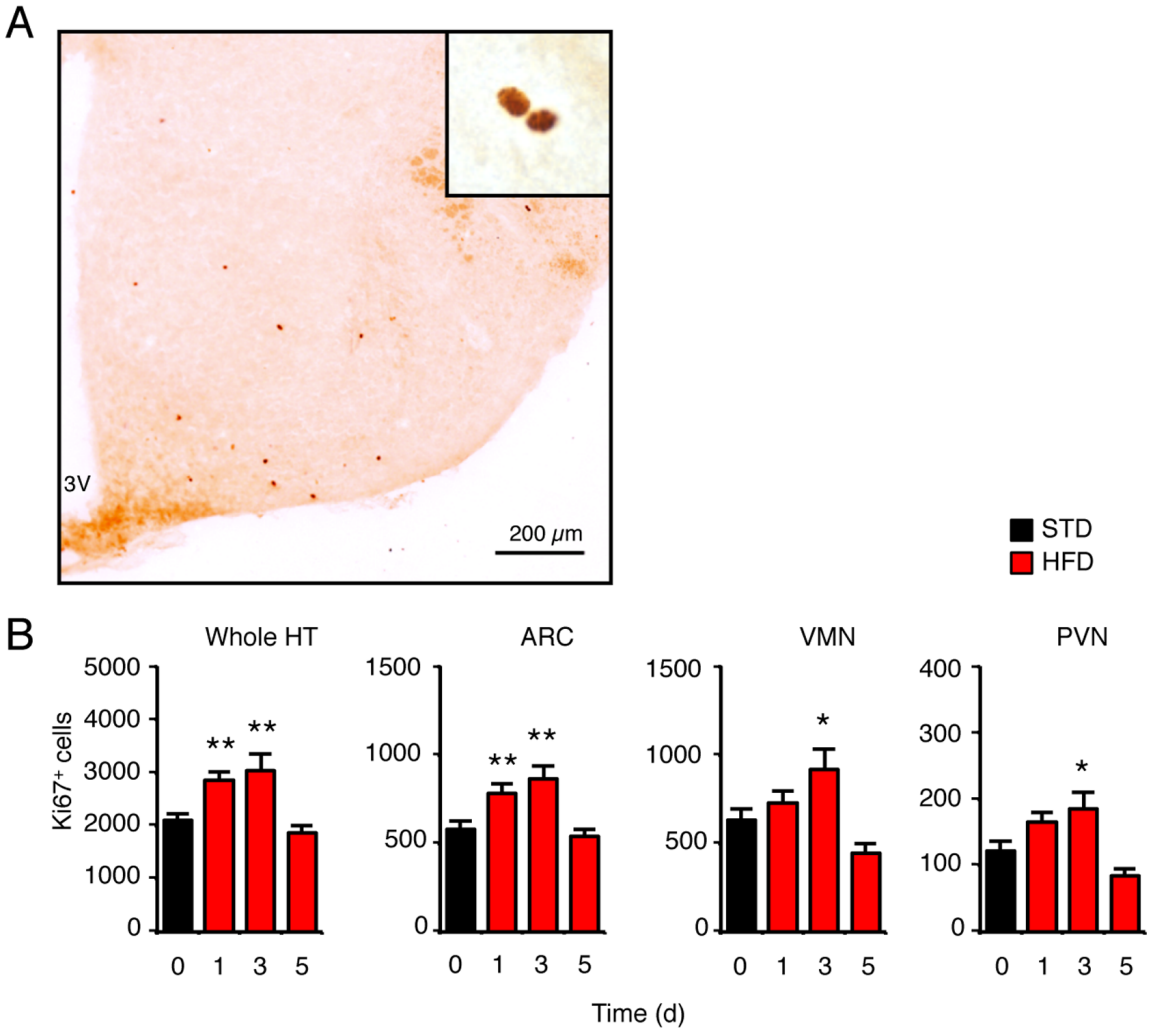
High-fat diet (HFD) transiently increased cell proliferation in the hypothalamus. (A) Representative image of Ki67-positive proliferating cells immunodetected in the hypothalamus of control mice. (B) Quantification of Ki67-positive cells detected in the whole hypothalamus and in selected hypothalamic areas in mice fed either a standard or HFD for 1, 3 and 5 days. Basal amount of Ki67-expressing cells at day 0 corresponds to the value found in control mice fed a standard diet. Data are means ± SEM (n = 12 at day 0, and n = 5 at day 1, 3, and 5). Groups were compared using ANOVA followed if positive by Newman-Keuls post-hoc test. * and ** denotes p≤0.05 and p≤0.01, respectively. 3V: third ventricle; Arc: arcuate nucleus; HFD: high-fat diet; PVN: paraventricular nucleus; STD: standard diet; VMN: ventromedial nucleus.

The increased accumulation of BrdU-positive cells observed in the hypothalamus of adult mice as soon as 2 days after HFD introduction could be the consequence of an enhanced cell proliferation. We thus quantified proliferating cells in animals that were sacrificed at 0, 1, 3 or 5 days after HFD introduction using immunohistostaining of Ki67, a marker expressed during all phases of the cell cycle except G0 (Fig. 2A). Control mice exhibited 2099±118 Ki67-positive cells in the whole hypothalamus (Fig. 2B). This result was consistent with the previously calculated value with linear regression for accumulation of BrdU-positive cells. In HFD mice, the number of Ki67-positive cells was increased at day 1 (2848±169; p<0.05 relative to STD) and day 3 (3035±323; p<0.05 relative to STD), but returned to control values at day 5 (1857±145; p>0.05 relative to STD). Therefore, these data indicated that HFD acutely stimulated cell proliferation within the hypothalamus. This was in agreement with the data obtained with BrdU infusion. To test whether HFD-induced cell proliferation occurred in hypothalamic nuclei involved in the control of energy homeostasis, we counted Ki67-positive cells in the arcuate nucleus (Arc), the ventromedial nucleus (VMN), and the paraventricular nucleus (PVN) (Fig. 2C). The number of Ki67-positive cells was increased at day 1 in the Arc of HFD mice (STD: 576±49 vs HFD: 782±51; p<0.05). At the same time, Ki67-positive cells were also higher in VMN and PVN of HFD mice, but these effects did not reach statistical significance. Nevertheless, the number of Ki67-positive cells was significantly increased in all the tested hypothalamic nuclei when mice were fed with HFD for 3 days. No difference was seen at 5 days. Thus, HFD stimulated cell proliferation in the hypothalamus during the first three days. This effect was first detected in the Arc and it later occurred in the VMH and the PVN.

The number of Ki67-positive cells in the dorsal vagal complex (DVC) of the brainstem was also slightly increased in HFD mice at day 1 (STD: 465±37 vs HFD: 527±23; p>0.05), and the difference became significant at day 3 (STD: 405±14 vs HFD: 616±59; p<0.05). In the subgranular zone (SGZ) of the hippocampus, we found about 8500 Ki67-positive cells in STD mice. No alteration was detected in this area after 1 or 3 on HFD.

### Accelerated Weight Gain Under HFD after the Blocking of the Brain Cell Proliferation

**Figure 3:**
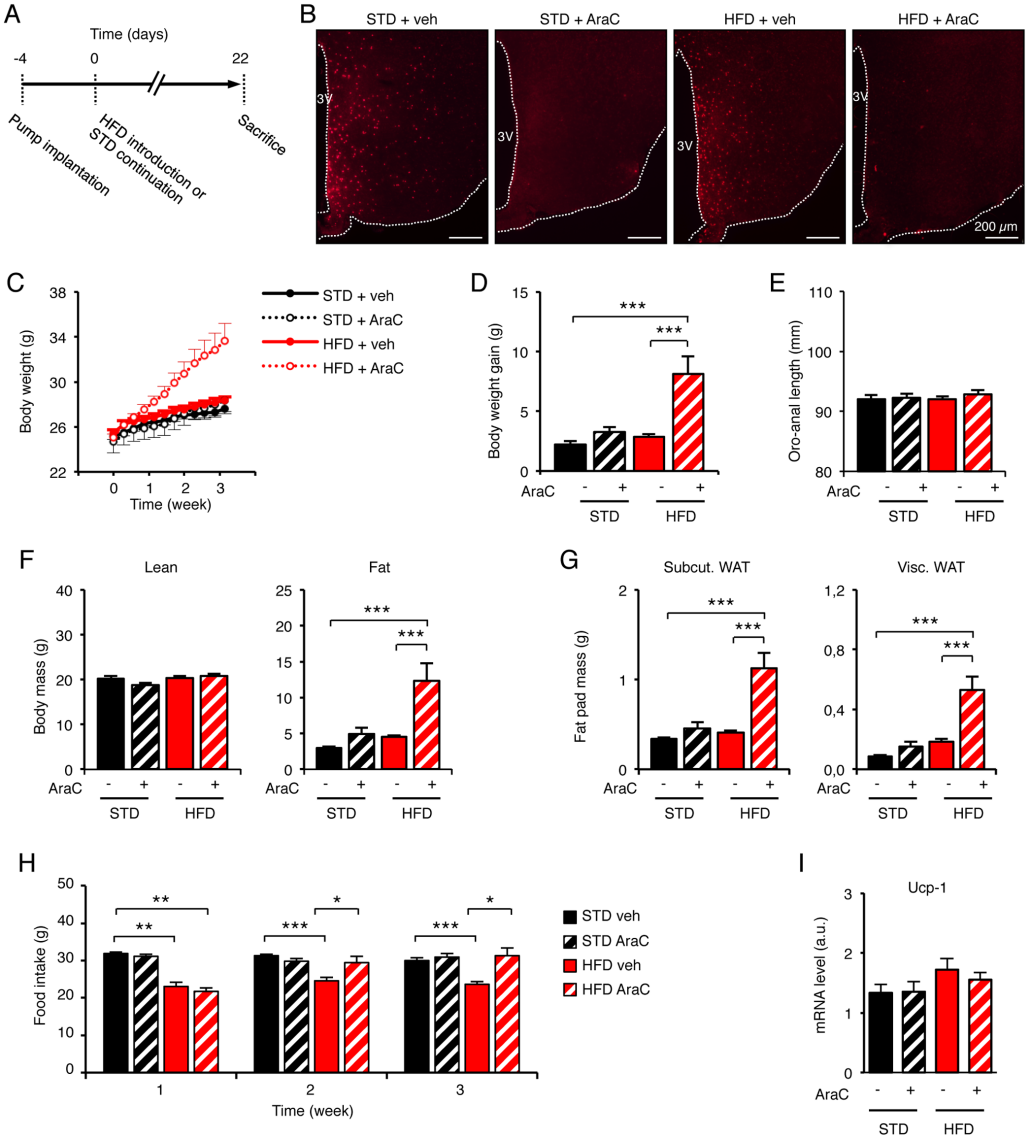
Blocking of the cell proliferation in the adult hypothalamus abolished the homeostatic feeding reponse to dietary fat and causes dramatic weight gain. (A) Protocol used to inhibit cell proliferation in the brain. AraC-filled osmotic minipumps were implanted subcutaneously, and connected to the ventricular system to centrally deliver 40 µg/day AraC (at 0.25 µl/hr) for 3 weeks. Some mice also received BrdU 6 µg/day through the same route. Food intake and body weight were monitored during the time-course of the experiment, whereas body weigth gain, oro-anal length, adiposity, mass of fat depots and Ucp-1 expression were determined at the end of the treatment. (B) After 22 days, brains were analyzed by immunohistochemistry against BrdU to assess the efficiency of AraC treatment. (C) AraC increased body weight of HFD-fed mice from 8 days and after, in comparison to all other groups. (D–F) AraC dramatically increased body weight gain of mice fed with HFD for 3 weeks in comparison to all other groups. This effect did not affect the animal growth, but did increase their adiposity. (G) Masses of both subcutaneous (Subcut) and viscerous (Visc) fat pads were similarly increased by AraC. (H) From the second week after HFD introduction, AraC inhibited the continuation of the homeostatic reduction of food intake. (I) Levels of Ucp-1 mRNA in the brown adipose tissue assessed by RT-qPCR. No difference were found between groups, suggesting that the higher food intake in AraC-treated HFD-fed mice was not compensated by induced facultative thermogenesis. Data are means ± SEM (n = 12–15, except for fat pads weighing: n = 5 per group, and for Ucp-1 analysis: n = 8–9 per group). Groups were compared using ANOVA followed if positive by Newman-Keuls post-hoc test. *, **, *** denotes p≤0.05, p≤0.01, and p≤0.001 respectively. 3V: third ventricle. AraC: Arabinofuranosyl Cytosine; HFD: high-fat diet; STD: standard diet; veh: vehicle; WAT: white adipose tissue.

To assess the role of neoformed cells on energy homeostasis, we permanently blocked the production of new brain cells in adult mice by central administration of the anti-mitotic arabinofuranosyl cytosine (AraC) as previously detailed [12]. Body weight and food intake were monitored during three weeks in mice kept on standard diet or HFD (Fig. 3A). AraC (40 µg/day) was continuously delivered through the osmotic minipump connected to the brain ventricular system. BrdU (12 µg/day) was co-administered in some animals to check the efficiency of the cell proliferation interruption. The post-mortem examination of brains by immunohistochemistry revealed that the AraC treatment totally inhibited the apparition of neoformed BrdU-positive cells in the hypothalamic parenchyma in standard and HFD mice as well (Fig. 3B). Nevertheless, the chronic AraC delivery did not alter the body weight of control animals (STD+veh: 27.69±0.44 g vs STD+AraC: 29.03±0.73 g after 3 weeks; p>0.05) (Fig. 3C, 3D), suggesting that the dosage and the procedure did not produce any toxic effect, as previously reported [12]. In contrast, the body weight of HFD animals treated with AraC was increased in comparison to the other groups. These mice became significantly heavier as soon as 8 days after HFD introduction in comparison to HFD mice treated with vehicle (HFD+veh: 26.89±0.29 g vs HFD+AraC: 28.22±0.54 g; p<0.05) (Fig. 3C). After three weeks on HFD, the body weight gain of mice treated with AraC was more than 3-times higher than the one of vehicle-treated mice (HFD+veh: +3.06±0.21 g vs HFD+AraC: +10.38±1.72 g; p<0.05) (Fig. 3D). No difference was seen regarding the body length of animals (Fig. 3E). At the end of the AraC treatment, the body composition was examined. AraC treatment significantly increased the fat mass of HFD animals (HFD+veh: +4.49±0.26 g vs HFD+AraC: +12.27±2.49 g after 3 weeks; p<0.05) (Fig. 3F), affecting both subcutaneous and visceral fat depots (Fig. 3G). Thus, blocking brain cell proliferation accelerates the onset of diet-induced obesity in mice, suggesting that cell renewal in the adult hypothalamus could exert protective functions against excessive body weight gain under detrimental nutritional conditions.

We also examined food intake regulation in AraC-treated mice receiving the standard diet or HFD (Fig. 3H). AraC treatment did not affect the basal food intake (i.e. around 30 g per week) in control mice under the standard diet at any of the examined time points. The introduction of HFD lead to a decrease in food intake during the first week that was similar in both mice treated or not with AraC. In HFD mice receiving vehicle, food intake was still reduced after 2 or 3 weeks of HFD (Fig. 3H). On the other hand, food intake increased in HFD mice treated with AraC, reaching levels similar to that observed in control animals. Therefore, AraC treatment prevented the long-term reduction of food intake that normally occured in HFD animals, suggesting that the cell renewal in the adult hypothalamus participated in the long-term regulation of food intake.

We finally examined the expression of Ucp-1, as a marker of thermogenesis, in brown adipose tissue (BAT). At the end of the experiment, total RNA were extracted from BAT biopsies and the abundance of Ucp-1 mRNA was analyzed by qPCR. The expression of Ucp-1 was similar in all groups. This suggested that the increased in food intake in AraC-treated HFD mice was not compensated by a higher energy expenditure through adaptive thermogenesis.

### Astroglial Cells as Part of the Proliferative Response to HFD

**Figure 4:**
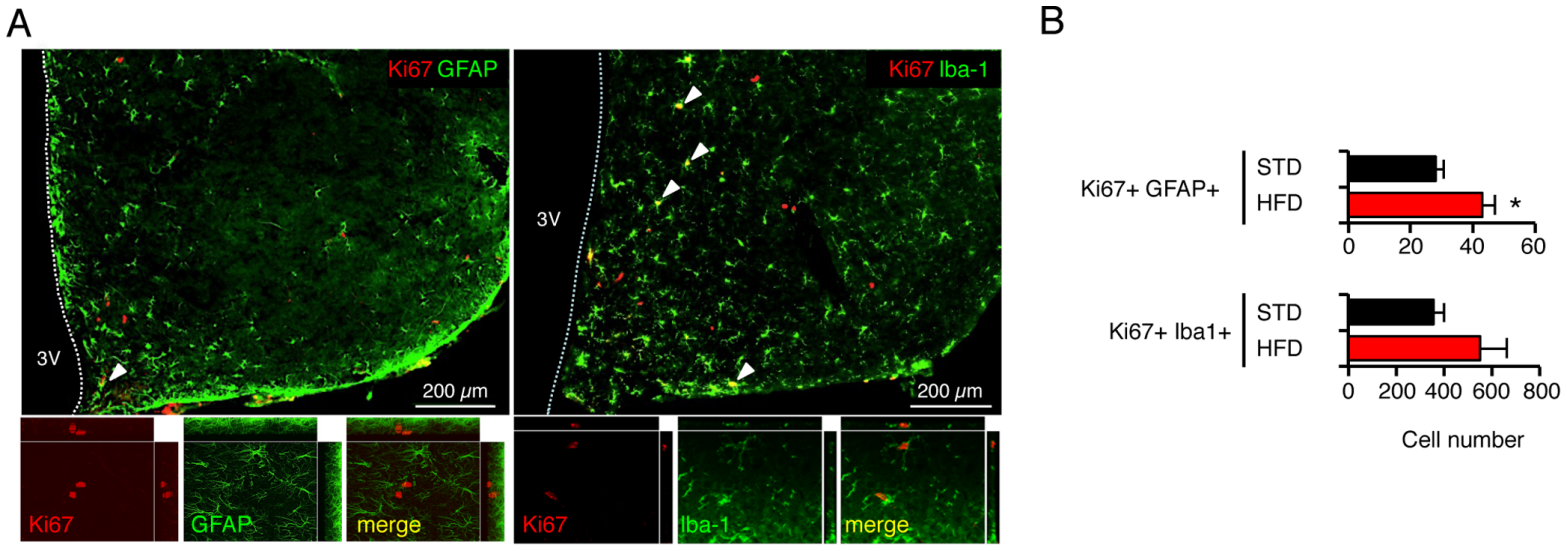
High-fat diet (HFD) for three days increased the pool of astroglial cells in the hypothalamus. Mice were kept on standard or HFD for 3 days and were then euthanized. Brain sections were examined after immunostaining of Ki67-expressing proliferative cells. (A) Representative images showing Ki67-positive proliferative cells (in red) detected in the hypothalamus and phenotyped by the following markers (in green): GFAP (astrocytes) and Iba-1 (microglia). (B) Quantification of double-labeled cells in hypothalamus of mice fed standard diet or HFD. Data are means ± SEM (n = 4–5 for each group). Groups were compared using unpaired t test. * and ** denotes p≤0.05 and p≤0.01, respectively; 3V: third ventricle. HFD: high-fat diet; STD: standard diet.

Our data indicated that brain cells generated during HFD acted to prevent subsequent body weight gain. To get insights into the cellular modalities underlying this anti-obesity effect, we investigated the phenotype of newborn cells during the time-course of HFD. We focused on the analysis of the hypothalamus since modifications in cell proliferation were detected in this brain area upon HFD. In addition to neural progenitors, numerous cell types exhibit proliferative properties in the adult hypothalamus, including astrocytes, tanycytes, microglia, and endothelial cells [3], [4], [6], [9], [18]. In order to identify the phenotypes of cells that proliferate in response to HFD, we used double immunofluorescence-labeling with antibodies against Ki67 to detect dividing cells, and against GFAP and Iba-1 to detect astrocytes and microglia respectively, in animals sacrificed after 3 days of HFD (Fig. 4A). In the whole hypothalamus of control animals, the number of positive cells was 28±3 for Ki67-GFAP and 354±45 for Ki67-Iba-1 (Fig. 4B). Thus, in the adult hypothalamus of mice, proliferative cells include glial cells. In the whole hypothalamus of HFD animals, these numbers were 43±4 for Ki67-GFAP, and 550±113 for Ki67-Iba-1. A significant increase in the number of Ki67-GFAP-positive cells (p<0.05, relative to control mice) and to a lesser extent in the number of Ki67-Iba-1 was found in HFD mice, indicating that HFD rapidly stimulated the proliferation of glial cells in the hypothalamus as previously described [19].

### No Modification of the Neuronal Fate of Newborn Cells under HFD in the Adult Hypothalamus

**Figure 5:**
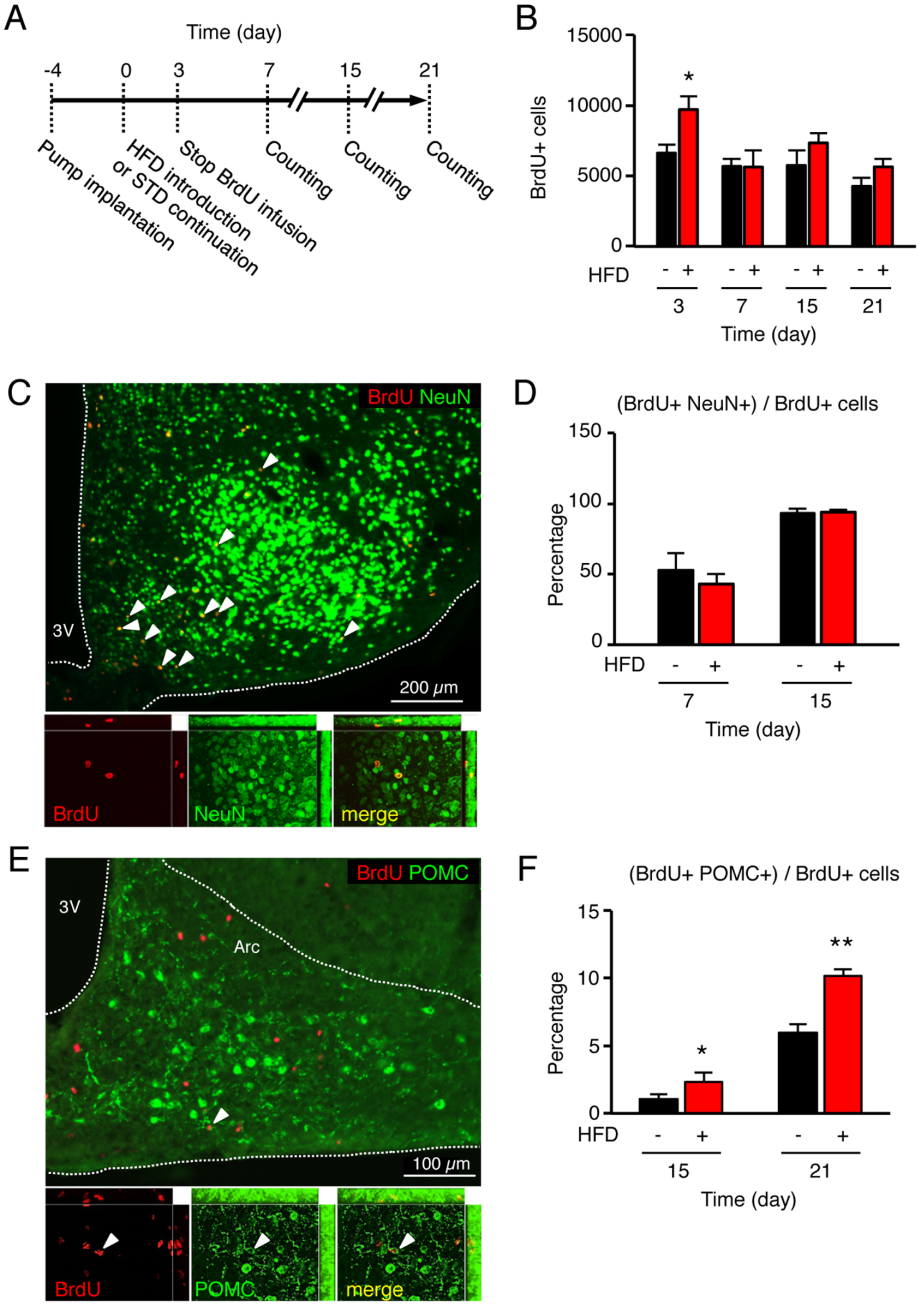
High-fat diet (HFD) did not deviate the neuronal fate rate, but incited new neurons to mature into anorectic POMC cells. (A) Schematic presentation of the experimental design. Four days before HFD introduction, BrdU-filled osmotic minipumps were implanted subcutaneously and connected to ventricular system to centrally deliver 12 µg/day BrdU (at 0.5 µl/hr). Infusion was stopped at day 3 by cutting the cathether. Mice were kept for an additional 1 to 3-week period and then euthanized. Brain sections were examined after immunostaining of BrdU-positive neoformed cells. (B) HFD did not alter the survival rate at 3 weeks of BrdU-labeled neoformed cells in the hypothalamus in comparison to control mice. (C–D) HFD did not alter the differentiation rate into neurons in comparison to control mice, as assessed by double immunohistostaining against BrdU (in red) and NeuN, a neuronal marker (in green). (E–F) HFD increased the number of arcuate newborn POMC neurons, as assessed by double immunohistostaining against BrdU (in red) and POMC precursor (in green). Representative images were obtained from control mice at day 21. Data are means ± SEM (n = 5–6 per group). Groups were compared using unpaired t test. *, **, denotes p≤0.05 and p≤0.01, respectively. 3V: third ventricle; Arc: arcuate nucleus; HFD: high-fat diet.

After 3 weeks on HFD, the food intake was still higher in mice treated with AraC compared to vehicle-treated mice. To test whether the long-lasting anorectic effect without AraC could be relayed by newborn neurons, we assessed the survival and the fate of newborn BrdU-positive cells in the hypothalamus at 1, 2 and 3 week(s) after HFD introduction (Fig. 5A). Mice were implanted with osmotic minipumps to centrally deliver BrdU, which were removed three days after HFD introduction. Thereafter, newborn cells were labeled with BrdU exclusively during the HFD-induced proliferative period (Fig. 5A). In control mice, the number of BrdU-positive cells in the entire hypothalamus progressively decreased from 6594±662 at day 3, to 4234±654 at day 21 (Fig. 5B). Thus, approximately 35% of the newly generated cells observed at day 3 were eliminated over the subsequent weeks in normal conditions. A similar trend was observed in HFD mice, where the number of BrdU-positive cells decreased from 9706±934 at day 3 to 5644±568 at day 21, reflecting a 40% elimination rate over the examined 3-week period. However, the decline in newly generated cells occurred much more rapidly in HFD mice. Indeed, the numbers of BrdU-positive cells were already strongly reduced at day 7 compared to day 3 in HFD animals vs control animals (5627±1172 vs 5653±541 BrdU-positive cells at day 7, respectively), representing an elimination rate of 40% vs 15% of the newly generated cells respectively. These data suggested that HFD strongly accelerated hypothalamic cell renewal, without altering long-term survival of newly generated cells.

The phenotype of BrdU-positive cells was then examined by double immunofluorescence-labeling using an antibody against the neuronal nuclei marker (NeuN) to reveal mature neurons (Fig. 5C). After 1 week, 52.6±12.0% of the BrdU-positive cells were also stained with NeuN in control mice (Fig. 5D). This proportion was of 93.4±2.8% after 2 weeks, indicating that the majority of newly generated cells in the adult hypothalamus differentiated into neurons in control conditions. In HFD mice, NeuN-BrdU-positive cells corresponded to 43.1±7.4% and 94.0±1.9% of total BrdU-positive cells, after 1 and 2 week(s) respectively, being thus similar to what occurs in standard conditions. Therefore, these data indicated that HFD did not alter the ability of newly generated cells to differentiate along the neuronal lineage.

### Increase in the Production of Arcuate POMC Neurons under HFD in the Adult Hypothalamus

Newly generated neurons in the adult hypothalamus could further maturate into numerous specialized neuronal subtypes that compose this brain region. Since AraC treatment increased weigh gain upon HFD, we thought that much more anorectic newborn cells could be generated as an adaptive process to further limit excessive energy intake on a long term. Among them, arcuate pro-opiomelanocortin (POMC) neurons plays a fundamental role in the regulation of appetite, and the adaptive metabolic response to an excessive caloric consumption [20], [21]. To test whether newborn cells differentiated into arcuate POMC neurons, BrdU-positive cells located in the arcuate nucleus were further characterized using an antibody against the POMC precursor peptide (Fig. 5E). Among total arcuate BrdU-positive neoformed cells, POMC-BrdU-positive cells represented 1.1±0.3% after 2 weeks, and 6.0±1.1% after 3 weeks in control mice (Fig. 5F). In HFD mice, this proportion was significantly higher (p<0.05 vs control), reaching 2.3±0.3% after 2 weeks and 10.2±0.6% after 3 weeks. Thus, a greater number of newborn cells maturated into POMC neurons in the arcuate nucleus of HFD mice, suggesting that the fate specification of POMC neurons was nutrionally regulated.

## Discussion

### Characterization of the Constitutive Cell Proliferation in the Adult Hypothalamus

Using chronic brain delivery of BrdU combined with post-mortem immunohistochemical analysis of BrdU-labeled cells, we characterized the cell turn-over of the murine adult hypothalamus. We found that approximatively 2,000 new cells were generated every day in the adult hypothalamus. By comparison, cell renewal in neurogenic structures of the adult forebrain is much important, 5 times more in the subgranular zone (SGZ) of the hippocampal dentate gyrus [22] and 15 times more in the subventricular zone (SVZ) of the lateral ventricles [23]. We further revealed that the majority of newly generated cells differentiated into NeuN-positive cells, confirming previous studies indicating the constitutive neurogenesis in the hypothalamus of adult mice [3], [6], [7], [11], [12], [14]. The post-natal neurogenesis in this area appears to be widely conserved throughout species [8], [9], [24], [25], [26]. We observed that the hypothalamic cell proliferation occured in the ependymal layer of the third ventricle, as it was previously described [2], [3] and in the parenchyma as well, in agreement with others [6], [7], [12]. Newborn neurons found in the adult hypothalamic parenchyma are thought to derive from tanycytes, the hypothalamic radial glia like-cells located in the ependymal layer [2], [3]. Moreover, doublecortin (Dcx), a protein found exclusively in migrating neurons [27], has been observed in scattered cells in the hypothalamus [6], [28], suggesting that immature neurons could migrate through the hypothalamus before their proper maturation. Regarding the fate of adult neoformed hypothalamic cells, we found that nearly all surviving cells after three weeks differentiate into neurons and a small fraction (only 6%) further maturated in POMC cells in the arcuate nucleus. Although the kinetics of the differenciation process of POMC neurons has not been established yet, *in vivo* neurogenesis in the mediobasal hypothalamus occurs very slowly under normal physiology conditions [14]. The constitutive neurogenesis in the adult hypothalamus might serve to replace degenerative cells [7] and to adapt neuronal circuits to ever-changing environmental and physiological conditions [9], [29].

### Stimulation of Hypothalamic Cell Renewal upon HFD

The main finding of the present study related to the effect of dietary fat on hypothalamic cell renewal. We demonstrated that HFD markedly and transiently increased cell proliferation in the hypothalamus as soon as 24 hours after diet introduction. This resulted in a massive accumulation of newly generated cells within 3 days of exposure to HFD. So far, responses to exogenous mitogens were investigated in the hypothalamus after three days or more, but never earlier [2], [4], [12], [13]. Likewise, the proliferation of neural progenitors has been evidenced after five days in heat-acclimated rats [9]. Our results suggested that the physiological regulation of neurogenesis in the hypothalamus exerted by dietary fat was a particularly fast event. The HFD-stimulating effect on cell proliferation was transient, lasting only three days, and was followed by a brutal drop of the neorformed cell number. The rapid disappearance of such a large number of newly generated cells is puzzling and the mechanisms at play here remains to be investigated. A possibility is that many of the newly generated cells underwent apoptotic cell death almost immediately after birth, while others survived. Similar cascade of events leading to the selection of newborn cells has been found during learning and sensory experience in early life [30], [31]. Taken together, these data suggested that newly generated cells in the hypothalamus undergo apoptosis in response to lack of trophic factor support, like in other brain regions subjected to adult neurogenesis [30]. Interestingly, HFD seemed to strongly accelerate the kinetics of these events compared to control animals, without altering the long-term persistence of BrdU-labeled cells. Thus, in agreement with studies describing adaptive physiological responses to increased dietary fat [15], [32], [33], this suggested that HFD could acutely mobilize adaptive processes at the cellular level as well.

The HFD-induced stimulation of cell proliferation was visible throughout the hypothalamic parenchyma, even if the arcuate nucleus was affected first. Previous studies have also reported a widespread distribution of BrdU-positive cells in the hypothalamus when pharmacologically stimulated [2], [4], [12], [13]. Hence, the regulation of neural progenitor proliferation in this area was not obviously restricted to any particular nucleus. Despite this apparent lack of regional specificity within the hypothalamus, the proliferative response to HFD might be site-specific at the level of the whole brain. Actually, the proliferative response to HFD appears restricted to brain areas that directly control appetite and metabolism, such as the hypothalamus and the dorsal vagal complex of the brainstem, since the dentate gyrus of the hippocampus, a highly proliferative brain area, was not affected in this time frame.

### Requirement of the Stimulated Cerebral Cell Proliferation to Limit Weight Gain upon HFD

To assess the function of newborn cells in energy homeostasis, we globally inhibited cell proliferation in the brain using the antimitotic AraC. This drug was administred chronically as previously described [12]. Remarkably, AraC treatment had no effect in mice under standard diet, but accelerated the onset of obesity when energy balance was disrupted by HFD introduction. This suggested that HFD-stimulated cell proliferation was involved in the homeostatic response to dietary fat, whereas constitutive cell renewal might not exert a fundamental function in energy balance regulation. On the other hand, long-lasting inhibition of constitutive cell renewal (i.e. more than three weeks) might have also impaired energy balance. Indeed, the constitutive cell renewal in the hypothalamus seems to be required to regenerate neuronal networks during neurodegeneration [7]. Site-specific manipulation of cell proliferation is technically challenging [3] and the intracerebroventricular route that we used to abolish brain cell proliferation makes hard to determine whether the hypothalamus is the only contributor to the observed response. However, our data converged at hypothalamic cell renewal as being primarily involved in the homeostatic response to HFD. This is consistent with the well-demonstrated role of the hypothalamus in the long-term regulation of energy balance. Because stimulation of cell proliferation upon HFD also occurs in the dorsal vagal complex of the braistem, another reactive neurogenic brain area [34], [35], it is likely that several brain areas that sense metabolic cues and control energy balance could be involved in the homeostatic response to HFD too.

We showed that the stimulated cerebral cell proliferation was required to limit weight gain upon HFD. Physiological processes affected by the AraC treatment during HFD consumption such as meal size and frequency, adaptive thermogenesis or intestinal assimilation were not determined yet. It is therefore difficult to conclude whether the HFD-stimulated cerebral cell proliferation exerted a direct control or not on the adiposity. Above all, the inhibition of HFD-stimulated brain cell proliferation by intracerebroventricular infusion of AraC increased weight gain, as well as blocking CNTF-induced hypothalamic cell proliferation by the same procedure [12]. On the other hand, the selective inhibition of neurogenesis in the adult median eminence by focal tomography-guided irradiation reduced weight gain of HFD-fed mice [3]. Taken together, these data suggested that the brain cell proliferation may have different effects on the body weight depending on its location.

### Hypothesis Regarding the Biological Mechanisms Underlying the Metabolic Effect of HFD-stimulated Cerebral Cell Proliferation

The mechanisms underlying the contribution of proliferative cells to the homeostatic response to dietary fat have not been established here. AraC caused overweight in HFD-fed mice after only one week. This time frame is hardly compatible with an effect requiring functional integration of newly generated neurons into existing neural circuits. Newborn neurons are not integrated into circuits before several weeks actually [36]. Hence, another mechanism should be considered in the model. We hypothesized that dividing cells including immature neural progenitors and reactive glia, could secrete anorectic factors within the first week after HFD introduction, and that this secretion could be involved in the rapid anorectic adaptive response. In this line, a surge of secreted mediators coming along with proliferation of glial cells was demonstrated a few days after HFD introduction [19]. Some of them are growth factors and cytokines, which act locally in the hypothalamic parenchyma. In fact, both the neural progenitors [37], [38], [39] and the reactive glia [19], [40] could secrete such biological mediators. Interestingly, some of the extracellular cues that promote neurogenesis such as insulin-like peptides, BDNF and CNTF, also reduce food intake and body weight [12], [41], [42]. A variety of cytokines share the same functions [43], [44], [45]. Reactive glia regulates adult neurogenesis [43] and later selection and integration of newborn neurons [46], [47], [48]. Hence, we propose that a mixture of extracellular factors could be secreted by proliferative cells in the hypothalamic parenchyma early on after HFD introduction and that these released factors could have paracrine effects on the hypothalamic cell proliferation rate itself and on neuronal systems that control body weight too. Thus, limiting HFD-stimulated proliferation by AraC could decrease the concentration of some of the secreted factors emitted by newborn cells, then altering both the amplification of the proliferative response and the acute secretome-dependent homeostatic response on the body weight.

This study suggested that HFD strongly accelerated hypothalamic cell renewal, without altering the long-term survival of newly formed cells. Moreover, these data indicated that HFD did not alter the ability of newly generated cells to differentiate along the neuronal lineage. Our immunostaining revealed a greater number of newborn cells maturated into POMC neurons in the arcuate nucleus of HFD-fed mice, suggesting that the fate specification of POMC neurons was nutritionnally regulated. The time required for functional integration of these new neurons and whether they are sensitive to metabolic cues remain to be elucidated. Nevertheless, our data suggested that the cell renewal was accelerated in response to the energy imbalance. Thus, it could be possible that newly generated hypothalamic neurons may integrate into existing networks more rapidly than the constitutive rate. Therefore, once functional, additional anorectic POMC neurons could be an ultimate defense against metabolic imbalance, to reinforce the HFD-induced up-regulation of POMC expression [32] and the HFD-induced synaptic plasticity of POMC neurons [15]. Although functionality of newborn POMC neurons remains to be determined, these results further confirmed that the melanocortin system was highly plastic and adapted to the nutritional conditions by a combinaison of various strategies. However, this physiological process was obviously inhibited when the calorific pressure was sustained [11], [14].

Overweight, which is dramatically rising in the world, is a major risk factor for a number of chronic diseases, including diabetes and cardiovascular diseases. A better characterization of the function of adult hypothalamic neurogenesis in the control of energy balance could help finding novel approaches to fight against obesity. In this study, we showed that adult hypothalamic neurogenesis was remarkably responsive to the nutritional conditions. We provided a precise description of the early cellular events engaged in the homeostatic response to dietary fat. Moreover, we showed that a defect in cerebral cell renewal lead to severe overweight, providing new insights into our understanding of nutritional regulation of energy balance.
